# Discovering DNA Methylation, the History and Future of the Writing on DNA

**DOI:** 10.1007/s10739-022-09691-8

**Published:** 2022-10-14

**Authors:** Joshua D. Tompkins

**Affiliations:** Arthur Riggs Diabetes Metabolism and Research Institute, City of Hope, 1500 E Duarte Road, Duarte, CA 91010 USA

**Keywords:** DNA methylation, Conrad Hal Waddington, Gerard Wyatt, Riggs and Holliday, Treat Johnson, Epigenetics

## Abstract

DNA methylation is a quintessential epigenetic mechanism. Widely considered a stable regulator of gene silencing, it represents a form of “molecular braille,” chemically printed on DNA to regulate its structure and the expression of genetic information. However, there was a time when methyl groups simply existed in cells, mysteriously speckled across the cytosine building blocks of DNA. Why was the code of life chemically modified, apparently by “no accident of enzyme action” (Wyatt 1951)? If all cells in a body share the same genome sequence, how do they adopt unique functions and maintain stable developmental states? Do cells remember? In this historical perspective, I review epigenetic history and principles and the tools, key scientists, and concepts that brought us the synthesis and discovery of prokaryotic and eukaryotic methylated DNA. Drawing heavily on Gerard Wyatt’s observation of asymmetric levels of methylated DNA across species, as well as to a pair of visionary 1975 DNA methylation papers, 5-methylcytosine is connected to DNA methylating enzymes in bacteria, the maintenance of stable cellular states over development, and to the regulation of gene expression through protein-DNA binding. These works have not only shaped our views on heritability and gene regulation but also remind us that core epigenetic concepts emerged from the intrinsic requirement for epigenetic mechanisms to exist. Driven by observations across prokaryotic and eukaryotic worlds, epigenetic systems function to access and interpret genetic information across all forms of life. Collectively, these works offer many guiding principles for our epigenetic understanding for today, and for the next generation of epigenetic inquiry in a postgenomics world.

## The Writing on DNA

Ever since the non-random distribution of four DNA “letters” was connected with the specific assembly of twenty unique amino acids—which emerged alongside the rise of information technology and was no doubt influenced by religious and scriptural notions of the “Book of Life”—DNA has been described as a deterministic code (Kay 2000). Molecular biologists, philosophers, and the general public have pondered the implications of rewriting the encrypted messages. Hollywood bustles with characters who have unlocked the fantastical secrets of genetic potential. The reality of biomedical progress, on the other hand, includes only a mere handful of examples of targeted sequence changes able to cure human disease and reduce suffering (Frangoul et al. 2021; Sharma et al. 2021). Nonetheless, a coded perspective of DNA is precisely what led to these first-generation cures and their anticipated expansion. In targeted patient cells, a pinpointed correction to the amino acid rendered in a pathogenic protein can be achieved, not by targeting the protein, but by altering the original site of the relayed information, the DNA. Still, a gene-centric code is an oversimplification of DNA and all the potential information it may harbor. In our current postgenomic era, new DNA concepts and language are continually needed to describe the complexities of DNA expression, especially during pivotal moments of development and disease (Fox 2002).

Although a genetic disease caused by a single point mutation does appear to be deterministic (DNA change = disease), and we can predictably change the sequence-determined outcome by genetic surgery, most letter changes in DNA are generally accepted within the cell without altering behavior, pathophysiology, or fate. In this regard, either most of the primary sequence—the *genome*—is an evolutionary byproduct, in which non-coding sequences are non-deterministic and formed from repetitive virus like depositors of “junk” DNA over time, or one must consider most fate changes at a systems level of genome regulation.

An enormous gap remains between genotype and phenotype, but in the age of *epigenomics* we have begun to witness the stochastic outcomes of developmental signaling in organisms with vast cell numbers arising from a single fertilized egg (Guo et al. 2017; Pastor et al. 2016; Tompkins et al. 2016; Xie et al. 2013).^1^ Here, the genome behaves nothing like physicists’ original notions of DNA as a static book of readable code (Fox 2002). Rather, it moves and wriggles with information, more akin to an optical fiber with countless potential and interconnectable branches of tunable light, some of which fold back into the primary cable. The issue for decoding is that each region of the genome is far from binary (either “on” or “off”), and gene “branches” represent only a tiny portion of the entire read sequence (ENCODE Project Consortium 2012). Further, the emergence of phenotype is not restricted to cellular information provided from within the cell and manifests on the multicellular level over time. Therefore, one must consider the auxiliary forces that reside above, or *epi-genetically* to, the primary DNA sequence as it is iteratively accessed for information.

DNA is enzymatically written upon. Chemically decorated at unique sites, gracefully strung around adjustable protein units, and dynamically organized into domains and territories within the greater nucleus, DNA appears to function as a complex information storage and retrieval system, at least to most contemporary biologists. The packaging is exquisite, and in select locations DNA is said to be *expressed*; single-stranded messages virtually identical to the copied bit of DNA are utilized to support the function or fate of a cell. As such, every cell has an *expression potential* that is inherent to the expressive metastable conformation—the optical network in our simile—of its genome. When a cell changes its identity, as in the case of cellular differentiation in development, it is reflected in DNA presentation: chemical decorations, DNA looping, and DNA expression appear to change in synchrony with cell fate. Are these *epigenetic* changes deterministic? Like the primary sequence, at the single epi-change level, a rare alteration may have profound consequences, but most will never alter the phenotype of an organism. Rather, it is systems level epigenome signaling over time and space, starting with simple embryo axis-polarity signaling of the totipotent egg, which initiates and constrains the induction of common organizing principles within the primitive embryo. This ultimately results in the same predetermined outcome generation after generation (that is, a new generation).

In this article, I reflect on a time when DNA was first discovered to be mysteriously written upon. As DNA purification strategies improved, it appeared that at least some of our genome was bonded with small alkyl groups consisting of three hydrogen atoms attached to a carbon. These *methyl groups* would eventually be found in bacteria, viruses, plants, and animals—across all life—but what did they do? How did we come to appreciate *DNA methylation* (DNAme) as a quintessential epigenetic force?^2^ In the following pages, I revisit key technical developments in biochemistry and biotechnology that drove this understanding. I also examine observations across multiple domains of life that informed the first concepts of how epigenetic modifications to DNA, like DNAme, could alter protein binding and access to primary genetic information. In a very real sense, when we witness chemical changes on DNA, we are watching the epigenetic coordination of genetic potential, the temporal and spatial access of genetic information within and across cells. We are also observing the accumulation of environmental history onto DNA. Why, where, and when is DNA naturally written on? Presently, we have achieved a better understanding of this process and related epigenetic factors that control DNA expression. Can we write our own messages on DNA? As of recently, the answer is yes.

## A Historical Introduction to Epigenetics

Although often amusing to many modern biologists, serious scientific debate surrounded preformationism until the late 1700s*.* Peering through early light microscopes, some naturalists believed they could see entire miniature humans elegantly packaged into sperm and eggs. According to theory, once fertilized, these so-called *homunculi* would simply grow to full maturation (Roe 1981). However, scientists would require that observations be subjected to repeat scrutiny, decade after decade, with an ever-increasing array of investigational tools and techniques. In time, and with better microscopes, the homunculus myth fell, and the “imagined embryo” replaced by the “observed embryo” (Maienschein 2003, 2014).

Aristotle’s concept of development as a homogenous egg material whose composition increases in complexity over development was refined by English physician William Harvey (1578–1657) around 1650. Through his studies on developing limbs, which were undoubtedly not preformed, as well as his discovery of the *cicatricula* in chick embryos (the yolk membrane area from which the embryo develops), Harvey effectively argued against preformation in favor of *epigenesis* (Deichmann 2016; Kilgour 1961). But what, then, was the enigmatic energy that directed developmental morphogenesis? If preformed anatomical structures were not inherited, how did physical and behavioral features emerge, one generation to the next?

In the early 1920s, Hilde Mangold (1898–1924), pursuing graduate studies with German embryologist Hans Spemann (1869–1941), conducted a series of newt blastopore transplantation experiments. In the few surviving recipient embryos, second neural tubes, brains, spinal cords, and heads developed, and in the course of time the two introduced a landmark concept in embryology: the *organizing center*. Also referred to as the “Spemann-Mangold organizer,” key groups of cells appeared to guide the fates of surrounding cells into increasingly intricate tissues (Spemann and Mangold 1924). British developmental biologist Conrad Hal Waddington (1905–1975) extended this concept into mammals with early embryo, primitive streak, and transplant experiments in chickens (Waddington 1932). In 1939, he first used the term *epigenotype* to describe “organizing relations to which a certain piece of tissue will be subject during development” (Waddington 1939, p. 156). Three years later, he further addressed this ominous gap in the understanding of developmental biology. “We certainly need to remember that between genotype and phenotype, and connecting them to each other, there lies a whole complex of developmental processes. It is convenient to have a name for this complex: ‘epigenotype’ seems suitable” (Waddington 1942, p. 10). Waddington went on to propose *epigenetics* as “the branch of biology that studies the causal interactions between genes and their products which bring the phenotype into being” (Waddington 1968, p. 5).

It is important to note that Waddington and contemporaries developed epigenetic concepts in an era of tremendous discovery about the basic structure and composition of DNA.^3^ In 1941, George Beadle (1903–1989) and Edward Tatum (1909–1975) developed what later was called the “one-gene-one enzyme” hypothesis, which described the initial flow of genetic information to a protein product (Beadle and Tatum 1941). In 1953, James Watson (b. 1928) and Francis Crick (1916–2004) famously solved the structure of the double helix of DNA, aided by X-ray crystallographic studies proposed by Maurice Wilkins (1916–2004) and successfully executed by Rosalind Franklin (1920–1958), coupled with the nucleotide pairing rules developed by Erwin Chargaff (1905–2002), for which they received a Nobel Prize (Watson and Crick 1953; Elson and Chargaff 1952; Franklin and Gosling 1953). Therefore, by the early 1950s, DNA was considered a replicating double helical structure present in all cells that stored information for making enzymes. The chasm between genotype and phenotype was narrowing, and the first complex systems of gene regulation had begun entering scientific imagination.

In 1958, the American geneticist David Nanney (1925–2016) articulated the state of the art, based on his study of the faithful inheritance of *Tetrahymena* mating-types: “The existence of phenotypic differences between cells with the same genotype merely indicates that expressed specificities are not determined entirely by the DNA present in the cell—that other devices, epigenetic systems, regulate the expression of genetically determined `potentialities`” (Nanney 1958, p. 713). That is, some “auxiliary mechanisms” must function to determine “which specificities are to be expressed in any particular cell” (Nanney 1958, p. 712). Around the same time, Waddington produced his celebrated “epigenetic landscape,” describing the restriction of developmental potential over time as a ball rolling downward through progressively narrowing and distinct valleys (Waddington 1957). Epigenetic barriers served to restrict developmental fate and reveal phenotype over time. Ultimately, Nanney and Waddington produced complementary epigenetic definitions in which genetic *specificities* (that is, DNA expression) are regulated by cellular factors that determine which “potentialities” are elicited over development.

Over the years, epigenetics has been used to describe both environmental factors that alter phenotype across populations and time and cellular factors that control gene expression over rounds of replication.^4^ For example, climate change has produced broad new selective pressures on animal populations, and epigenetic variation can be sensitive to major changes to the environment. This contributes to the flexibility of a population’s phenotypic response to climate change (Hu and Barrett 2017). Then again, the pollution that drives climate change can physically alter the chemical writing of methyl groups on our own DNA, disrupt normal gene expression, and, over time, drive the transformation of healthy cells into cancerous ones (Rider and Carlsten 2019). In the ensuing pages, I will predominately describe DNA methylation (DNAme) as it is concerned with the latter epigenetic picture. By understanding the discovery, history, and nature of DNAme—the literal attachment of chemical groups to DNA to regulate DNA expression and the maintenance of these patterns in stable cell fates—the function of epigenetics as a force that records environmental and developmental history onto DNA structures becomes mechanistically clear. Finally, these concepts will be considered in the postgenomic era—that is, the epigenomics and epigenetic engineering era—and used to define epigenetics for this transition in biology.

## The Discovery of 5-Methyl Cytosine (5mC)

By 1950 it was known that there were 4 letters constituting our genetic code, yet it was not yet understood how Adenine (A), Thymine (T), Guanine (G), and Cytosine (C) were connected, read, and replicated. For Watson and Crick, essential insight was provided when Yale biochemist Erwin Chargaff famously used partition chromatography on filter paper (*paper chromatography*) to separate and precisely analyze the constituents of nucleic acids from different cell types (Chargaff 1950). The separation of pyrimidines was carried out in aqueous butanol, and, after paper migration, the position of each unique DNA letter was visualized with ultraviolet (UV) absorption using a UV spectrophotometer. Chargaff observed approximately equal levels of A and T, or G and C, across species. So, if DNA was double-stranded, helical, and in accordance with hydrogen bonding between facing DNA letters, then A must always pair with T, and G with C. What was overlooked, however, was that Chargaff’s cytosine levels were slightly low relative to guanine (Chargaff 1950).^5^ Where was the missing cytosine?

The discrepancy was not enough to be mentioned in Watson and Crick’s model, but Chargaff had some competition: another work was cited in determining their base-pairing rules. Gerard Wyatt (1925–2019), who had recently graduated from the Molteno Institute, independently confirmed A and T levels were virtually identical within an organism. Furthermore, he could account for the missing cytosine among the paper chromatogram signals. The modified DNA letter closely resembled cytosine and, importantly, brought G-C ratios into “proper unity” (Wyatt 1950, 1951). Although this observation was not incorporated into the original model of the double-helix, for the first time Wyatt’s detection placed a modified DNA letter among the base pairing rules of the structure.

Before moving forward from Wyatt’s work, it is important to clarify who actually detected 5-methylcytosine (5mC) first. The discovery of DNAme is often attributed to American biochemist and geneticist Rollin Hotchkiss (1911–2004), when he detected methylated cytosine, calling it *epicytosine*, among hydrolyzed calf thymus samples (Hotchkiss 1948, p. 323).^6^ This was indeed the first detection of 5mC in higher eukaryotes, published a few years before Wyatt’s observations. Or, alternatively, articles have cited American biochemist Waldo Cohn (1910–1999) for his work on the adaption of ion-exchange chromatography to radioisotope label nucleic acids as part of the Manhattan Project (Cohn 1951). Like Hotchkiss, Cohn also detected 5mC in calf thymus extracts. However, Wyatt, Hotchkiss, and Cohn were not the first to detect 5mC. Rather, all cited decades-old research by American chemists Treat Johnson (1875–1947) and Robert Coghill of Yale (1901–1997).

**Fig. 1:**
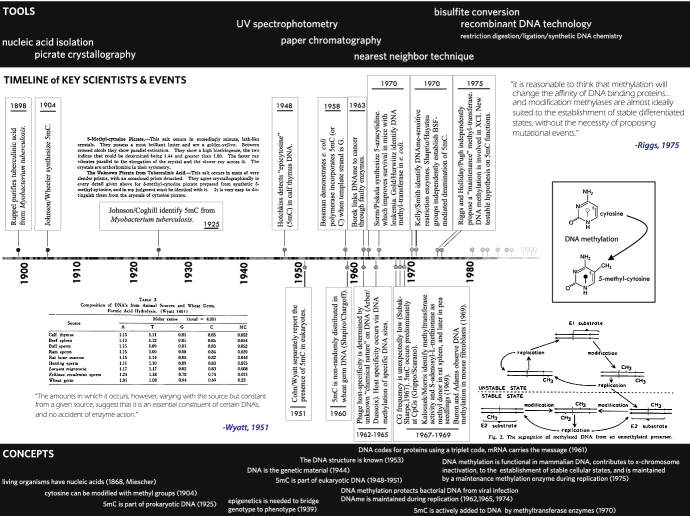
DNA methylation discovery, 1898–1975, with key tools, scientists, and concepts. A selection of the most important tools developed in DNA methylation analysis are listed above the timeline. These tools were employed by leading scientists of the time to create a progressively clearer picture regarding the nature and potential functions of methylated DNA, which parallel major breakthroughs in understanding DNA structure and in developing epigenetic concepts (bottom box). Additional details are included for the works of Johnson (1925), Wyatt (1951 and 1952 table), and Riggs/Holliday (1975), for the detection of 5mC in prokaryotes (and synthesis), the detection of 5mC in eukaryotes across species, and the proposed functions, maintenance mechanisms, and testable hypotheses in higher eukaryotes, respectively. The structure of cytosine and methylated cytosine (5-methyl-cytosine or 5mC) is displayed right, with the symbol for methylated cytosines represented as a closed circle. As with the distribution and overall impact of specific historical breakthroughs in DNA methylation research across the timeline, methylated cytosines are non-randomly distributed across DNA and have unique impacts on local levels of DNA expression. The timeline therefore also serves to illustrate this core epigenetic concept, in which a DNA backbone is decorated with methyl groups at some cytosines and can be read as a form of molecular “braille.” As a classical example, if a group of methylated cytosines exist near the beginning of gene, they are interpreted as instructions to promote stable gene silencing

It was Johnson, at the time working with Henry Wheeler (1872–1945), who published the original synthesis of 5-mC in 1904. The paper, aptly titled “5-Methylcytosine,” begins by noting that cytosine is converted to uracil in hot acid and suggests that 5mC might give thymine (5-methyluracil) in a similar manner (Johnson and Wheeler 1904). The structure of 5mC, with the methyl group appended to the 5′ carbon of the pyrimidine ring, is displayed in Fig. 1. Additional reasoning or discussion for this work is not given, which was among many papers Johnson published in organic chemistry. By the 1920s, Johnson’s goals, as related to biology, were more articulate:

There has been a missing link in a chain of chemical and biological evidence, which has prevented us from presenting a picture of nucleic acid-pyrimidine chemistry…. The introduction of a fourth pyrimidine, methyl-cytosine into our family of cell pyrimidines would enable us to complete our series…. [T]he next problem of immediate biochemical interest would be proving whether these aminopyrimidines [5mC] are primary structural units of the nucleic acid molecule. (Johnson 1925, p. 2841)

In other words, did 5mC exist in a living organism?

To answer this question, Johnson, working with Coghill, crystallized 5mC (and C) with picric acid for microscopic examination. Much like a fingerprint, the crystal is unique to each pyrimidine and derivative. Whereas cytosine crystals produced beautifully long needles and oblique crystal terminations, 5mC needles were small and displayed squared ends simple enough to visually distinguish. They next hydrolyzed tuberculinic acid (containing DNA) isolated from tuberculosis bacterial cultures and crystallized the pyrimidines. There, displayed among the crystal lattices, was the tell-tale signature of 5mC. It appears Johnson had long expected to detect this compound in living organisms. Writing in 1925, he stated:

Ever since the publication of this synthesis of 5-methyl-cytosine in 1904, the writer has anticipated the discovery of this pyrimidine among the products of hydrolysis of nucleic acid, but it was not until it was made possible for us to make an examination of the nucleic acid obtained from tubercle bacillus that … we now find this aminopyrimidine, 5-methyl-cytosine, is one of the products of hydrolysis…. [T]he discovery of this compound increases the number of pyrimidines functioning in life. (Johnson and Coghill 1925, p. 2841)

By the 1950s, Hotchkiss, Wyatt, Cohn, along with virtually every biological scientist, understood that DNA was the heritable material of life, but it remained unknown whether 5mC was part of eukaryotic life. If so, how and why? Armed with new, powerful tools in chromatography and the precedence of Johnson’s 1925 prokaryotic work, all three would detect eukaryotic 5mC in rapid succession (Hotchkiss 1948; Wyatt 1950, 1951, 1952; Cohn 1951). Neither Hotchkiss nor Cohn would speculate on the function of 5mC, stating only that “it is true that epicytosine stands in the same relation to cytosine with respect to both its absorption spectrum and its mobility in butanol that thymine (5-methyluracil) does to uracil. More than this cannot be said until further study of epicytosine has been made” (Hotchkiss 1948, p. 326). Wyatt’s work, on the other hand, was different in one significant way. Like Chargaff, Wyatt was examining ratios of the four primary DNA building blocks across many different species. Therefore, he did not simply detect the presence of this modified base in a single higher eukaryote. Rather, he observed 5mC in calves, bulls, rams, rats, herrings, locusts, sea urchins, and wheat germ (see Fig. 1). Levels of 5mC were extraordinarily high in plants and, importantly, always brought G-C ratios to unity.

Although Wyatt, like Hotchkiss and Cohn, stated that “nothing can be said as to the possible function of 5-methylcytosine,” it was clear that his observations had already implied function. His next sentence reads: “the amounts [of 5mC] in which it occurs, however, varying with the source but constant from a given source, suggest that it is an essential constituent of certain DNAs, and no accident of enzyme action” (Wyatt 1951, p. 583). That is, there was some reason for DNAme to exist, and there must be a basis by which the unique and constant DNAme levels are maintained across species.

It is uncertain as to why Wyatt failed to detect 5mC in tuberculosis, as Johnson and Coghill observed in 1925. He suggested that cytosine contamination of 5mC crystals may have disturbed Johnson’s work; however, subsequent reports have since confirmed 5mC within prokaryotic genomes, including *Myobacterium tuberculosis* (Wyatt 1951; Hemavathy and Nagaraja 1995; Srivastava et al. 1981; Rein et al. 1998). Ironically, it would be Wyatt who, a short time later, would detect yet another cytosine variant that currently is in the limelight for its unique functions in stem cells and neurons. Searching deeper in the prokaryotic world, hidden among the viruses that infect bacteria, 5′hydroxymethylcytosine also brought G-C ratios “close to unity” in bacteriophages (Wyatt and Cohen 1952). In discussing his collaborative work with biochemist Seymour Cohen (1917–2018) at Cold Spring Harbor, Wyatt wrote: “One is tempted to speculate that regular structural associations of nucleotides of adenine with those of thymine and of guanine with those of cytosine (or its derivatives) in the DNA molecule requires that they be in equal number” (Wyatt 1953, p. 780). Therefore, Wyatt was tantalizingly close to Watson and Crick’s work and, in fact, crossed paths with Watson at the Pasteur Institute. Robert Olby captured Wyatt’s description of their encounter.

Jim Watson was sitting on a high stool with his legs wrapped around it and was describing to us this wonderful idea that he and Crick had just had about the structure of DNA. It hit me immediately that it must be correct, their variation must follow a fixed pattern by which these ratios (A-T and G-C) are kept unchanged. (Olby 1974, p. 221)

Whereas Wyatt could “scarcely even speculate upon how this would occur,” Watson and Crick would accommodate this fixed pattern of repeating A’s, C’s, G’s, and T’s into their semi-conservatively replicating model of DNA. Soon, the theoretical incorporation of 5mC (and variants) into this structure would raise new questions about DNA binding and gene regulation, catalyze the development of new biotechnology tools, and solidify DNAme as an enduring field of epigenetic research.

## The Unexpected Distribution of 5mC

In the helix, if G is always positioned across from C, what then is the case for 5mC? In 1958, biochemist Arthur Kornberg (1918–2007) and colleagues answered this question. *E. coli* DNA polymerase was indifferent in positioning 5mC across from G, and therefore it is unlikely that 5mC’s were uniquely distributed by DNA polymerase during DNA replication (Bessman et al. 1958). Rather, specialized enzymes for methylating DNA must exist. Kornberg shared the challenge ahead: “what I shall present is that the replication of DNA can be examined and at least partially understood at the enzymatic level even though the secret of how DNA directs protein synthesis is still locked in the cell” (Kornberg 1959, p. 665). The answers to this secret, however, were already in the works and represent important precursors to sufficiently understanding DNAme as an epigenetic element. A few years earlier, researchers had first begun witnessing traces of ribonucleic acid (RNA) to rapidly form in bacteria following bacteriophage infection (Cobb 2015). To explain how this RNA molecule resembled the phage’s DNA, François Jacob (1920–2013) and Jacques Monod (1929–1976) famously named the intermediate of genetic information “messenger RNA.” Subsequent experiments with Sydney Brenner (1927–2019) and Matthew Meselson (1930–) confirmed phage-linked mRNA to be associated with ribosomes, which established a definitive connection between DNA and protein synthesis (Brenner et al. 1961; Jacob and Monod 1961; Cobb 2015). Ultimately, mRNA fit perfectly into the forming “Central Dogma” of molecular biology (Crick 1958), and so it was that DNA is transcribed into RNA, which is translated into protein.

Besides major insights into DNA replication, Kornberg’s group also developed a powerful *nearest neighbor* technique for determining DNA nucleic acid composition. This technique involves detection of radioactive phosphate when it is transferred from one DNA letter to its nearest neighbor, enabling determination of the frequency of any two bases occurring together (AA, AT, AG, etc.). Standing out among dinucleotide pairs, cytosine followed by guanine (“CpG,” where p represents the phosphate bond between letters) was uniquely depleted among vertebrate genomes (Josse et al. 1961; Swartz et al. 1962). The reduction was approximately fivefold less than would be expected to occur by random chance. Seven years later, Pasquale Grippo and Eduardo Scarano, working at the University of Palmero with sea urchins, established that CpG dinucleotides harbored a surprising 90 percent of all 5mC (Grippo et al. 1968). These prominent CpG features hold true for our genome as well, which relates to the spontaneous deamination of 5mC in genomes (ultimately to thymine) (Greenberg and Bourc'his 2019). In CpG methylated genomes, like those of vertebrates, evolutionary pressure has driven most CpG’s out of existence, but where they do exist, they are most likely modified by DNAme. If one imagines a long stretch of DNA, where modifications are restricted to rarer CpG locations that are non-randomly distributed, then we might imagine a basic epigenetic structure. Here, DNAme functions as a form of “molecular braille,” where it is sensed and interpreted by proteins that “read” DNA and express its information as RNA in some locations (Fig. 1).

If something exists on DNA, there must have been a biological or chemical factor that interacted with DNA and left a mark. Therefore, researchers next sought out the enzyme responsible for the strange methyl groups being discovered on DNA. In 1963, working at the Albert Einstein College of Medicine, Jerard Hurwiitz (1928–2019), along with Marvin Gold and Monika Anders, observed DNA methyltransferase activity in bacteria. The enzymatic function relied on S-adenosylmethionine as a methyl donating molecule (Gold et al. 1963). Again, building on observations from prokaryotes, researchers at Rutgers would next establish methyltransferase activity from rat spleens and pea seedlings (Kalousek and Morris 1969a, b). Therefore, as predicted by Wyatt in 1951, it appeared that 5mc was in fact deposited “by no accident of enzyme action.”

## The Proposed Maintenance and Function of 5mC

During the 1960s and early 1970s, the building blocks for recombinant DNA technology were being uncovered, and these would also have an important role to play in understanding how DNAme actually functions. Bacteria appeared to employ specific enzymes capable of digesting viral DNA and restricting infections. Across the prokaryotic world, an immense range of these “restriction enzymes” were identified, cataloged by the unique DNA sequence they digested, and co-opted to customize DNA fragmentation for recombination with other fragmented DNAs (Smith 1979). Some DNA digesting enzymes were observed to be uniquely sensitive to methylated sequences. Originally predicted by Swiss microbiologist Werner Arber (1929-) in 1965, bacteria harbored DNA digesting enzymes that could selectively recognize and cut invading phage DNA based on their lack of protective host DNA modifications (Arber 1965). Therefore, DNAme appeared to function as part of a basic phage recognition mechanism to protect bacteria (Meselson and Yuan 1968). Further, this observation suggested that methylated DNA might alter the DNA binding affinity of an enzyme.

By 1970, microbiologist Hamilton Smith (1931-) and his post-doctoral fellow Thomas Kelly (1941-) had purified the first type II restriction enzyme (Kelly and Smith 1970), and the gluing of the residual “sticky ends” by DNA ligase completed all basic features of a recombinant DNA reaction (Loenen et al. 2014). Scientists would use recombinant DNA technology to negotiate with bacteria in their circular plasmid DNA language by inserting segments of linear DNA into the temporarily opened plasmid of bacteria. First, recombination of bits of the galactose regulating sequence with SV40 of lambda phage was performed by David A. Jackson and Robert Symons (1934-2006) in Paul Berg’s (1926-) laboratory in 1972; then, more complex functional plasmid designs were carried out by Seymour Cohen (1917–2018) and Herbert Boyer (1936–) in 1973. Boyer would next target the multifaceted regulation of human insulin in bacteria (Jackson et al. 1972; Cohen et al. 1973).

Heading towards the biotechnology revolution, the first system of gene regulation was revealed. In 1961, Jacob and Monod, building on their mRNA work and studying lactose metabolism in bacteria, established that some individual genes could be regulated together. Many of their mutant bacteria had altered lactose-responsive gene expression levels, but there was no change in the enzyme function itself. Surprisingly, these mutations consistently mapped not to the coding sequence for the enzyme but rather to a nearby segment of DNA that functioned like a control center (Jacob and Monod 1961). This suggested that DNA expression could be regulated by protein binding at regulatory regions of DNA located near genes. By understanding the so-called *lac operon* and working closely with Herbert Boyer at University of California-San Francisco, City of Hope researchers Keiichi Itakura (1942–) and Arthur Riggs (1939–2022) coupled synthetic DNA chemistry with restriction digestion and ligation to produce two key recombinant DNA products. First, the simpler hormone somatostatin and, second, insulin, ultimately destined to save millions of diabetic lives.^7^

To produce a human gene product such as insulin within a prokaryote, it is insufficient to simply introduce the insulin gene in circular form. Rather, one must control the timing of insulin expression, delaying production until after enough bacteria have been replicated. To this end, Riggs was the first to purify the lac repressor to homogeneity, and he used it to regulate insulin expression. It was during these studies on protein–DNA interactions that Riggs began to interweave concepts of differential protein-DNA affinity with the ideas of Susumu Ohno (1928–2000) regarding X Chromosome Inactivation (XCI). Ohno’s work, which had in large part inspired Riggs to join the City of Hope, described a phenomenon by which the second X-chromosome in females is condensed and silent in all somatic cells, despite having an identical DNA sequence to the active X chromosome (Ohno 1969; Ohno et al. 1959). Within an individual cell, how can one long strand of DNA be routinely utilized while an identical strand is simply carried forth, condensed and ostensibly silent, with each new round of cell division?

Ohno was also an accomplished evolutionary biologist, writing the renowned book *Evolution by Gene Duplication* (Ohno 1970). To him, some DNA appeared to exist “only for itself without contributing to an organism’s fitness” (Ohno 1972). Over time, these repeating and self-copying segments may simply have represented scattered evolutionary “junk.” In contrast, Roy Britten (1919–2012) at the Carnegie Institute and Eric Davidson (1937–2015) at Caltech proposed that gene regulation may occur through multiple types of genes that interact with one another. This assigned a potential function to at least some of these repeating non-coding DNA sequences (Britten and Davidson 1969). Their ideas of “sensor” genes were visionary, far preceding today’s notions of regulatory elements that function to govern gene expression levels. Importantly, Britten and Davidson emphasized the requirement that some genes must be regulated by select DNA binding events, but suggested that this was determined by the primary sequence itself. To Riggs, XCI’s permanence and randomness early in development, arising from two chromosomes of the same DNA sequence, required an additional conceptual level to gene regulation.

As Riggs stated,

Eukaryotic methylation has not been discussed in light of the recent evidence accumulating about the E. coli lac repressor and DNA methylases, nor has emphasis been placed on the advantages of methylation for permanent changes in regulation occurring during differentiation. (Riggs 1975, p. 20)

He went on:

5-Methylcytosine also should effect regulatory protein binding because it is well established that the lac repressor is very sensitive to minor changes in the major groove…Therefore, it is reasonable to think that methylation will change the affinity of DNA-binding proteins for their binding sites … and modification methylases are almost ideally suited to the establishment of stable differentiated states, without the necessity of proposing mutational events. (Riggs 1975, pp. 17, 20)

In other words, DNAme likely controls protein-DNA interactions that facilitate gene regulation and XCI. As for the inactivation of one X-chromosome, “Randomness follows easily if the critical methylation event takes place after fertilization,” and “the differentiated state resulting from methylation is erased whenever DNAme is prevented during replication … during oogenesis or spermatogenesis, or during the rapid DNA replication cycles following fertilization” (Riggs 1975, p. 19). The “advantages” of DNAme are that it functions epigenetically without altering the DNA sequence itself, connects the selective DNA binding of bacterial methylases to general transcription factor function, and allows for both permanent and reversible gene expression states.

In the same paper, Riggs remarkably proposed both the existence of and mechanisms for a maintenance DNAme enzyme. He understood that if DNAme is required for establishing stable cellular states, then a copying mechanism for similar cell types must exist. Independently, molecular biologists Robin Holliday (1932–2014) and his student John Pugh had come to similar conclusions and were putting forth roles for DNAme in forming “developmental clocks” during ordered embryonic development. They proposed a virtually identical maintenance methylation mechanism to that of Riggs. Here, an enzyme we now understand to be DNA methyltransferase I (Dnmt1) preferentially recognizes and methylates hemi-methylated DNA during DNA replication (Fig. 1) (Holliday and Pugh 1975).^8^ In this way, existing DNAme patterns are sensed, copied, and maintained cellular-generation-to-generation.

Although neither Riggs nor Holliday and Pugh explicitly used the term *epigenetic* in these landmark papers to describe DNAme’s role in gene regulation and in forming stable cellular states, the conceptual connections with contemporary epigenetic definitions are clear. Over the next several decades of study, “DNA methylation” would become nearly synonymous with “epigenetics” and would be accompanied by an ever-growing list of epigenetic features that were also capable of influencing genome expression. Basic histone modifications were observed in 1968 by pioneers Vincent Allfrey (1921–2002) and Alfred Mirsky (1900–1974) (Vidali et al. 1968), and clusters of DNA wrapped histones would be defined as *nucleosomes* shortly thereafter, 1973–1975 (Olins and Olins 2003). Therefore, the next decades in molecular biology and genetics were filled with example after example of epigenetic factors that could alter the presentation of DNA without changing the sequence. The genome would emerge as something quite different from a double-helix with a few DNA binding proteins: it would come to resemble more an interactive 3-dimensional communication network, where protein–RNA–DNA interactions loop DNA upon itself into intricate, shape-shifting, and expressive conformations. If we organize changes to DNA expression over time and in response to developmental signals, a molecular basis for Waddington’s ball and for a forming embryo intuitively follows.

Using DNA methylation discovery, Riggs and Holliday provided a launching point for this future understanding: “Nevertheless, in view of our almost complete ignorance of the mechanisms for unfolding of the genetic program during development, it seems justifiable to suggest speculative hypotheses that may lead to meaningful experimental approaches” (Holliday and Pugh 1975, p. 231). Riggs also made a further suggestion: “Many of the points emphasized in this paper are, at least in principle, experimentally testable” (Riggs 1975, p. 20). In the end, by consolidating many important observations regarding bacterial methylating systems and applying these concepts to developmental biology and gene regulation, heritability was connected to DNAme, mechanisms of DNAme maintenance described and new testable hypotheses on the deposition and protein-DNA binding effects of DNAme put forward. These would catalyze decades of epigenetic discovery, and scientists continue the work to this day.

## Peering at Postgenomics Through the DNA Methylation Lens

In their quest to understand DNA methylation, scientists used prokaryotic discovery to guide eukaryotic investigation. For example, if methylated DNA exists in both prokaryotic and eukaryotic worlds, then the molecular machinery necessary for copying and adding new methyl groups to DNA must exist in both. This has turned out to be true, and genomics has informed us that essential sequences are conserved over evolution. However, despite the often-intimate proximity and coevolution of these worlds, the higher order complexity of eukaryotic DNA requires seemingly endless epigenetic devices to package and express such complex assemblages. Currently, we remain in a time of genome-wide association studies (GWAS) to inform us where distinct protein and RNA complexes are temporally and spatially located to regulate the genome within a variety of cell types and states.

Why is a genome expressed in the manner it is? Why does this expression vary over time? Why are some cells in stable states, others more plastic and capable of differentiation, and yet others pathological? These are primary questions of the epigenomic era left unanswered by genomics*,* highlighted by the use of GWAS on vast scales.^9^ This postgenomics era specifically involves the complexities of characterizing thousands, if not millions, of individual epigenomes in multiple cell and tissue types across different ethnicities, sexes, ages, and diseases. Such large-data inquiry remains necessary to understand how epigenetic nature, disease propensity, and behaviors may shift in populations over time (Richardson 2015). Therefore, in many ways we are still answering the same questions Waddington, Nanney, Riggs, and Holliday were originally asking and merely understand the dynamic nature of DNA and encoded potentialities a little more. Immense sets of epigenomic data remain to be generated and deciphered, and, as with past DNAme discoveries, a few new tools are likely to push us even further.

Among these, single-cell epigenetic and expression profiling technologies have already been particularly fruitful in defining local roles for specific epigenetic marks (Guo et al. 2017; Stelzer et al. 2015; Li et al. 2018). Whereas this technology is coming into fuller maturation, the capacity to accurately time, write, and erase one’s own epigenetic features is likely to drive the next phase of genome inquiry. Recently, an old encounter in the prokaryotic world has riveted the globe and transformed genetic research. Discovered in 1987, Clustered Regularly Interspaced Short Palindromic Repeat (CRISPR) sequences of DNA were observed to be strangely interjected across *E. coli*’s genome (Ishino et al. 1987). Much like the odd distribution of CGs in our genome implied some undefined functional role, why would bacteria harbor clusters of repeating inverted DNA sequences? And why did these, like so many bacterial DNA features, resemble bacteriophage DNA?

The answers to these questions culminated in the 2020 Nobel Prize, in which the revolutionary works of Emmanuelle Charpentier (1968-) and Jennifer Doudna (1964-) demonstrated CRISPRs to stem from an adaptive bacterial immune system. CRISPR DNA was observed to be transcribed as *crispr* or *guide* RNAs, which would direct the sequence-specific recruitment of a CRISPR associate protein (CAS) to target invading bacteriophage DNA for destruction (Hille et al. 2018). The CRISPR repeats, it appeared, functioned as a physical DNA “memory” of a preceding phage infection, which could be used to protect the bacteria against a secondary infection. An amazing war between bacteria and phages was indelibly written within their genomes, and the prokaryotic world harbored proteins that could be delivered to digest specific double-stranded DNA sequences with simple RNA instructions. In 2013, modified CRISPR/CAS systems were introduced into eukaryotic cells (Cong et al. 2013). Almost any DNA sequence, in virtually any species, can now be directed for digestion and therefore edited (Jinek et al. 2012; Cong et al. 2013). In only a few years, in vitro genome editing has become somewhat routine for most genetic and molecular biology laboratories.

## Engineering DNA Methylation

If one can recruit a Cas protein to cut or nick DNA for sequence editing, what happens if the enzyme’s cutting capacity is removed? First executed in 2013, researchers rendered a Cas enzyme (dCas) “dead” by mutating both of its DNA endonuclease nicking domains. The resulting protein then resides at a user-defined genome sequence, which is then ready for epigenetic modification (Qi et al. 2013). With the ability to routinely deliver dCas to virtually any DNA sequence, this platform represents the first truly genome-wide capable epigenetic-editing approach: the physical fusion or recruitment of an epigenetic modifying protein or protein complex builder to a dCas protein, bound to a target DNA sequence, to locally change epigenetic marks. For example, dCas can be fused to DNA methyltransferase to site-specifically alter DNAme and gene expression (Vojta et al. 2016).

Although this is immensely powerful in concept, there are evident limitations. Across the approximately 2 m of DNA folded inside each of our cells, how does one coordinate simultaneous epigenetic modification to multiple locations both efficiently and accurately, using a large bacterial protein that may or may not be delivered to a particular cell? Which genomic locations are amendable? When should this be done? Can large open or condensed chromosomal regions, spanning thousands or even millions of DNA letters, be changed with a protein that has a “footprint” of only around 75 base pairs (Josephs et al. 2015)?

CRISPR/Cas has already proven to be the type of technology that drives conceptual innovation (Creager and Landecker 2009; Vora et al. 2016; Cong et al. 2013). It has fundamentally altered how scientists study many local genetic and epigenetic changes in their native environment. This has enabled direct functional editing of known and putative epigenetic modifying proteins and RNAs for study and potentially for use in future therapies (Vora et al. 2016; Lo and Qi 2017). It is expected that CRISPR/Cas systems will inform and guide many forms of epigenetic investigation for decades.

However, dCas-based recruitment strategies are researcher-designed adaptations of a naturally occurring targeted DNA digesting device. Other prokaryotic and eukaryotic systems exist that are decidedly more epigenetic in nature. If history is our guide—specifically DNAme history—then we should expect some of these systems to remain undiscovered and that in due time basic observations will lead to the deciphering of these systems, refine our understanding of epigenetic mechanisms, and eventually catalyze the development of new tools for the next generation of DNAme researchers.

## DNA Methylome and Epigenome Engineering in the Postgenomics Era

In this article, I refer to a time when scientists puzzled over the simple existence of methylated DNA in prokaryotes. Why, again, were some prokaryotic sequences similar in composition to phage DNA and specifically methylated? Some enzymes appeared to be sensitive to DNAme: bacterial restriction enzymes could be used to sense unmethylated invading phage DNA and digest this DNA. Therefore, bacteria have immune systems, and enzymes that can methylate DNA must and do exist. With the coevolution of our prokaryotic and eukaryotic worlds, not only do we have DNA methyltransferase enzymes, but perhaps it should not be too surprising that we also harbor our own phage-like DNA sequences. Previously regarded as “junk DNA,” these remnants of ancient viral infections and hopping DNA bits, so-called *transposable elements*, remarkably make up nearly half of our entire genome. The sequences are, for the most part, also DNA methylated (Jansz 2019). There are key exceptions: very early in development, late in aging, and in diseases like diabetes and cancer, we see loss of DNAme and pathological reactivation of ancient repetitive sequences (Du et al. 2016; Li et al. 2015; Skvortsova et al. 2019). Therefore, DNAme functions to repress repetitive elements, and there are developmental windows where this function is particularly dynamic.

With the dawn of whole-genome sequencing and DNA *methylome* sequencing technologies around the turn of the twenty-first century, a remarkable tissue-specific accumulation of DNAme has been observed over differentiation and in mammalian development (Jansz 2019; Greenberg and Bourc'his 2019; Stelzer et al. 2015; Tompkins et al. 2016).^10^ Such accumulation helps to establish global epigenetic programs that transcriptionally manifest cellular composition, behavior, and function. This type of somatic information must be eliminated before a new being can be generated, that is, the germline is epigenomically reprogrammed to a sort of “fresh start” for every developing germ cell (the primordial germ cell). This occurs again after the male and female pronuclei fuse post-fertilization. In the case of DNAme, demethylating systems result in virtually all loss of 5mC by the 16-cell stage of development. Loss of DNAme is associated with dynamic repetitive element expression, which may have regulatory roles early in embryogenesis, and drive evolution. At this stage, the embryo is then ready for tissue-specific epigenetic decorations over time and in response to developmental cues, where many DNAme marks are permanently written and iteratively built into development and many environmental exposures are recorded directly on DNA and into DNA packaging systems (Jansz 2019; Greenberg and Bourc'his 2019; Tompkins et al. 2016).

It is here, at the intersection of prokaryotic and eukaryotic defenses to viral infections—the suppression of the legacy of these infections in our germ line and in early embryos, and the dysregulation of these DNA elements in pathological states—that novel epigenetic factors are likely to be discovered and co-opted for greater potential in epigenomic engineering. In embryonic or pluripotent stem cells, which represent the earliest stages of development, insertion of CG-free DNA into large stretches of CG-rich DNA (that is, the CpG “island”), spontaneously induces DNAme of dozens to hundreds of cytosines in the surrounding region. The deposited marks are naturally maintained by DNA methyltransferases through cellular differentiation (Takahashi et al. 2017). Although the exact mechanism for this phenomenon remains to be identified, the stimulation of a methylation response to an inserted sequence early in development is reminiscent of known mechanisms for suppressing transposable elements. Novel epigenetic editing tools, capable of larger scale DNAme edits, are likely to be found here. Further, a window for epigenetic manipulation exists before and within development, and some edits, relatively stable ones like DNAme, are likely to be maintained throughout life. In other words, Waddington’s landscape could, in principle, be “pre-contoured” prior to development and the epigenetic potential of a naïve cell tailored before development even commences.

Though one may be capable of operating on the epigenome through development, I caution against premature notions of tailoring Lamarckian inheritance. Much like infrequent sites of DNAme are retained in the germ line by imprinting mechanisms and some environmental exposures, particularly endocrine disruptors, induce rare DNAme events that can be transgenerationally inherited, ostensibly pinpointing DNAme edits early in development and carrying them forward into trillions of unique cells can hold unforeseen consequences (Kang et al. 2011). If, for example, we epigenetically “hide” a gene, whether permanently or temporarily, this is structurally distinct from excising or mutating the same genetic information and in some cases is expected to have unique phenotypic consequences.

A transition from site-specific epigenetic edits to systems level deterministic epigenomic manipulation appears even more daunting; it is true that we remain somewhat infantile in our understanding of developmental pleiotropy. Despite all our advances, the gap between genotype and phenotype still harbors incredible mystery, but this is a mystery that scientists and philosophers will never ignore. Therefore, we should recognize that an era of epigenomic guidance has naturally fallen on the heels of genomic manipulation, and with this will come all the same ethical considerations regarding any alteration to the germ line and/or early embryos. These concerns, in the context of human treatments, safety, and evolution, have been recently explored, including efforts towards a global moratorium on human gene editing (Doudna 2019). Developmental human epi-gene editing should also be under such consideration.

## Defining Epigenetics for Today

DNA methylation is a quintessential epigenetic mechanism. It is biochemically read and written to regulate genome presentation and remains perhaps the clearest example of how DNA can be selectively expressed. From the original synthesis of 5mC in 1904 to the direct DNAme editing technologies of today, the DNAme discoveries presented in this article have guided our ever-improving understanding of cellular and environmental factors that alter genome expression without changing the underlying DNA sequence. In reverence to this history and to a future of epigenome engineering, a comprehensive definition for epigenetics is offered.

**Epigenetics:** The cellular and organismal heritability of internal factors, including the modifications to them and by them, those recorded from environmental influences and in developmental history, whether physically local to the cell, signaled across an organism, or accumulated from sources larger in nature (for example, hormones, pollution, viruses, diet, and lifestyle), that influence the expression of chromosomally associated genetic information, establish stable cellular states over differentiation (or unstable states in aging and pathology), and enable the physical, biochemical, behavioral, cognitive, and social nature of an organism to emerge and function, without altering the primary DNA sequence.

## Data Availability

Not applicable.

## Abbreviations

5mC: 5-Methylcytosine
CpG: Cytosine-phosphate-guanine
DNAme: DNA methylation
GWAS: Genome-Wide Association Study

## Footnotes

1 Whereas the *genome* is the entire DNA primary sequence, the *epigenome* is the collection of all epigenetic factors that influence the genome presentation in each cell. In the *epigenomic age*, epigenomes are characterized for millions of cell types and states.

2 DNA methylation: the covalent addition of methyl groups to DNA by methyltransferase enzymes.

3 For background reading, see National Library of Medicine, Francis Crick Papers, “The Discovery of the Double Helix, 1951–1953.”. https://profiles.nlm.nih.gov/spotlight/sc/feature/doublehelix, Morange (1998), and Keller (2002).

4 For additional reading on original epigenetic concepts, see Deans and Maggert (2015); on contemporary misuse of the term, see Deichmann (2016).

5 The exception being in man: Chargaff detected equal levels of G and C levels within sperm, which we now understand to have very little cytosine methylation.

6 Of note, the naming of epicytosine is purely coincidental as related to *epigenetics*, in which the Latin prefix epi- simply means “on or above”.

7 For the fascinating story of how these scientists merged technical and conceptual innovations to introduce the first human-designed genes into bacteria that sparked the modern biotechnology industry, see Hall (2002).

8 *Hemi-methylated* refers to when one strand of DNA, the template strand during DNA replication, has a pre-existing DNA methylation pattern that has yet to be copied to the newly synthesized strand. DNMT1 prefers this structure for enzymatic activity and copies the methylation pattern to the new strand.

9 Whereas the genome is the entire DNA primary sequence, the *epigenome* is the collection of all epigenetic factors that influence the genome presentation in each cell. In the epigenomic age, epigenomes are characterized for millions of cell types and states.

10 DNA *methylome*: the total pattern of DNA methylation levels at all DNA sites which can be DNA methylated.
